# Triglyceride-glucose-body mass index predicts early-onset acute kidney injury in critically ill patients: a retrospective analysis using the MIMIC-IV database

**DOI:** 10.3389/fnut.2025.1721579

**Published:** 2026-01-12

**Authors:** Qiang Zhu, Qunchuan Zong, Shiying Guo, Yonghong Ma, Miao Zhang, Ningjing Jin, Yinggui Ba, Huajie Zou, Ruixia Zhang

**Affiliations:** 1 Department of Endocrinology and Metabolism, The Affiliated Hospital of Qinghai University, Xining, China; 2 Department of Endocrinology and Metabolism, Yibin Fifth People’s Hospital, Yibin, China; 3 Department of Traumatology and Orthopedics, The Affiliated Hospital of Qinghai University, Xining, China; 4 Department of Nephrology, The Affiliated Hospital of Qinghai University, Xining, China

**Keywords:** AKI, critically ill, insulin resistance, metabolic dysfunction, TyG-BMI

## Abstract

**Background:**

Acute kidney injury (AKI) is a common and serious complication in critically ill patients, with metabolic dysfunction playing a crucial role in its pathogenesis. The triglyceride-glucose-body mass index (TyG-BMI) has emerged as a novel marker of insulin resistance and metabolic health. However, the relationship between TyG-BMI and early-onset AKI in critically ill patients remains unclear. The aim of this study was to evaluate the association between TyG-BMI and early-onset AKI in critically ill patients, and identify optimal cutoff thresholds for risk stratification.

**Methods:**

This retrospective study analyzed 4,024 critically ill adults from the MIMIC-IV database. Patients were stratified according to TyG-BMI quartiles. Cox proportional hazards models, restricted cubic splines (RCS), and receiver operating characteristic (ROC) analyses were employed to examine associations between TyG-BMI and early-onset AKI. Optimal cutoff values were determined using the Youden index, while net reclassification improvement (NRI) assessed incremental predictive value.

**Results:**

Early-onset AKI developed in 2,535 patients (63.0%). Multivariable-adjusted hazard ratios increased across TyG-BMI quartiles, with the highest quartile showing significantly increased risk compared to the lowest (HR 1.40, 95% CI: 1.25–1.58). Risk increased approximately linearly when TyG-BMI exceeded 261.84. The optimal cutoff value was 252.50 (sensitivity 0.604, specificity 0.648). Adding TyG-BMI to traditional risk models improved prediction (NRI = 0.141, 95% CI: 0.024–0.207). Associations were stronger among males, younger patients, those with preserved eGFR, and patients with diabetes or sepsis.

**Conclusion:**

Triglyceride-glucose-body mass index independently predicts early-onset AKI in critically ill patients. The threshold of 252.50 offers a reliable reference for risk stratification in ICU settings.

## Introduction

Acute kidney injury (AKI) represents a significant clinical challenge in intensive care settings, affecting approximately 30%–50% of critically ill patients (1, 2) and is associated with substantially increasing mortality, hospitalization length, and risk of progression to chronic kidney disease (3, 4). Common causes of AKI in critically ill patients include sepsis, heart failure, drug-related nephrotoxicity, mechanical ventilation, post-major surgery status, and hemodynamic instability (5–8). Early identification of patients at high risk for AKI is crucial for improving outcomes through timely interventions and preventive strategies. Despite advances in critical care medicine, early recognition of patients susceptible to AKI remains problematic. Current tools for predicting AKI still primarily rely on traditional indicators such as baseline serum creatinine and urine output, which often demonstrate limited sensitivity and specificity across different ICU populations (9). Existing disease severity scoring systems [such as Sequential Organ Failure Assessment (SOFA) and Simplified Acute Physiology Score II (SAPS II)] can assess overall patient condition but are not specifically designed to evaluate AKI risk (10). Consequently, there is an urgent clinical need for novel, readily accessible markers that can effectively stratify AKI risk and facilitate timely preventive interventions. These limitations highlight the need for innovative predictive biomarkers that can complement existing tools and enhance early AKI risk stratification.

Recent research indicates that metabolic dysregulation plays a key role in the pathophysiology of AKI (11), particularly in critically ill patients. Alterations in lipid metabolism, glucose homeostasis, and inflammatory responses have been demonstrated to participate in kidney injury through various mechanisms, including endothelial dysfunction, oxidative stress, mitochondrial damage, and renal tubular epithelial cell injury (12, 13). These metabolic disturbances interact with kidney function in complex ways, therefore, developing integrated scoring systems that incorporate multiple metabolic indicators rather than relying on single parameters for AKI risk prediction and stratification is of paramount importance in critical care settings. The triglyceride-glucose-body mass index (TyG-BMI) integrates parameters of lipid metabolism, glucose regulation, and body composition, representing an emerging composite marker (14). This index can be easily calculated from routine laboratory tests (triglycerides and glucose) and basic anthropometric data (height and weight), requiring no specialized testing or additional costs. TyG-BMI has demonstrated predictive value for various cardiometabolic conditions, including insulin resistance (15), metabolic syndrome (16), and cardiovascular events (17, 18). Recent studies suggest that TyG-BMI may also be closely associated with kidney function deterioration, potentially through pathways involving systemic inflammation and microvascular dysfunction (19, 20). Although TyG-BMI has been extensively studied in chronic metabolic diseases, its application in acute critical care settings remains largely unexplored, particularly regarding AKI in critically ill patients. As a simple, readily available index, TyG-BMI has the potential to complement existing AKI prediction methods, providing clinicians with an easily implementable risk stratification tool in daily practice.

This study aims to address these gaps through three main objectives: (a) to evaluate the association between admission TyG-BMI values and subsequent development of early-onset AKI; (b) to identify optimal TyG-BMI cutoff thresholds for effective risk stratification; and (c) to examine potential effect modifications by key clinical factors, including diabetes status, obesity, and disease severity. Findings from this study may provide new insights for early risk stratification and targeted preventive strategies in critically ill patients.

## Materials and methods

### Study design and population

This retrospective cohort study analyzed data from 50,920 adult patients treated at Beth Israel Deaconess Medical Center (Boston, Massachusetts) between 2008 and 2019, using the publicly available MIMIC-IV database (version 3.0). The study protocol received approval from the Institutional Review Boards of the Massachusetts Institute of Technology (protocol 0403000206) and Beth Israel Deaconess Medical Center (protocol 2001-P-001699/14). Patient data were deidentified in compliance with the Health Insurance Portability and Accountability Act (HIPAA), and the requirement for informed consent was waived. The study adhered to the Declaration of Helsinki principles and followed Strengthening the Reporting of Observational Studies in Epidemiology (STROBE) guidelines.

We included patients aged ≥18 years who were admitted to the ICU and had complete baseline demographic information and essential laboratory measurements obtained within 24 h of ICU admission, including fasting triglycerides, fasting glucose, and serum creatinine. Additionally, documented height and weight were required for Body Mass Index (BMI) calculation. Exclusion criteria comprised: (1) pre-existing end-stage renal disease documented in medical history; (2) patients receiving chronic dialysis prior to admission; (3) patients who developed AKI before ICU admission based on Kidney Disease: Improving Global Outcomes (KDIGO) criteria (increase in serum creatinine by ≥0.3 mg/dL within 48 h, increase in serum creatinine to ≥1.5 times baseline within 7 days, or urine volume < 0.5 mL/kg/h for 6 h); and (4) patients with missing data on key variables necessary for TyG-BMI calculation or outcome assessment.

Baseline clinical data were systematically collected from the MIMIC-IV database and included comprehensive demographic characteristics (age, sex, ethnicity), detailed comorbidity profiles (identified using ICD-9 and ICD-10 codes), admission vital signs (blood pressure, heart rate, respiratory rate, temperature, oxygen saturation), comprehensive laboratory parameters (complete blood count, triglycerides, glucose, serum creatinine, blood urea nitrogen), and validated disease severity scores (SOFA score, SAPS II). All laboratory measurements were obtained within the first 24 h of ICU admission to establish accurate baseline values and minimize the influence of therapeutic interventions on the parameters of interest.

The MIMIC-IV database is publicly accessible to credentialed researchers who have completed a training course on human subjects research and signed a data use agreement. Access to the database is available through PhysioNet.^1^

### Exposure and outcomes

The primary exposure variable was TyG-BMI, calculated as ln[fasting triglycerides (mg/dL) × fasting glucose (mg/dL)/2] × BMI, with measurements obtained within 24 h of ICU admission. The primary outcome was early-onset AKI, defined as AKI occurring within 48 h of ICU admission according to KDIGO criteria, which include an increase in serum creatinine by ≥0.3 mg/dL within 48 h or an increase in serum creatinine to ≥1.5 times baseline within 7 days. Secondary outcomes included overall AKI during ICU stay and late-onset AKI (occurring after 48 h of ICU admission).

### Statistical analysis

Baseline characteristics of the study population were described using means with standard deviations (SDs) or medians with interquartile ranges (IQRs) for continuous variables, depending on their distribution, and as counts with percentages for categorical variables. The Shapiro-Wilk test was used to assess the normality of continuous variables. For comparisons, the Kruskal-Wallis test was applied to continuous variables, while the chi-square (χ^2^) test was used for categorical variables.

Cox proportional hazards models were used to calculate hazard ratios (HRs) and 95% confidence intervals (CIs), adjusting for baseline characteristics, including age, sex, estimated glomerular filtration rate (eGFR), severity at admission, and comorbidities. For multinomial logistic regression analysis of AKI severity, adjusted odds ratios (ORs) with 95% CIs were calculated for adjacent AKI stage comparisons.

The dose-response relationship between TyG-BMI and AKI was analyzed using restricted cubic splines (RCS). Likelihood ratio tests were conducted to evaluate non-linearity in the association between TyG-BMI and AKI. If no significant non-linearity was detected, linear associations were analyzed instead.

Predictive performance was assessed using receiver operating characteristic (ROC) curves with area under the curve (AUC) calculations. DeLong’s test compared AUCs between different outcomes. We determined optimal cutoff values using the Youden index and evaluated the incremental value of adding TyG-BMI to established risk models using net reclassification improvement (NRI). Decision curve analysis assessed clinical utility across various threshold probabilities.

Subgroup analyses were conducted to assess potential effect modification by age, sex, eGFR, SOFA score, and key comorbidities, with interaction terms included in the fully adjusted models to test the statistical significance of these interactions.

Missing data were handled using multiple imputation with chained equations for variables with <5% missingness.

Statistical analyses were performed using SPSS (version 27.0), Stata (version 18.0), R software (version 4.0.3) and appropriate packages. A two-tailed *p*-value < 0.05 was considered statistically significant.

## Results

### Baseline characteristics

The study included 4,024 critically ill adults (median age 64 years [IQR: 52–75]; 60.3% male). As shown in Table 1, AKI developed in 3,374 patients (83.8%), with 2,535 (63.0%) experiencing early-onset AKI and 839 (20.8%) developing late-onset AKI. Patients were stratified into quartiles based on TyG-BMI values: Q1 (≤217.16), Q2 (217.17–259.05), Q3 (259.06–314.75), and Q4 (≥314.76). Higher TyG-BMI quartiles were associated with significantly increased prevalence of Diabetes Mellitus (DM) and sepsis (both *p* < 0.001). The incidence of AKI differed across TyG-BMI quartiles, with overall AKI rates of 18.8% in Q1 and 22.9% in Q4 (*p* < 0.001). Early-onset AKI occurred in 12.7% of patients in Q1, increasing to 19.3% in Q4 (*p* < 0.001), while late-onset AKI decreased across quartiles (*p* < 0.001).

**TABLE 1:** Clinical and metabolic parameters for critically ill patients according to quartiles of TyG-BMI.

Characteristics	TyG-BMI				
	≤217.16	217.17–259.05	259.06–314.75	≥314.76	*P*-value
Number of participants	1006	1006	1006	1006	
**Demographic**					
Age (years)	67 (54–80)	67 (54–78)	63.5 (53–74)	60 (50–69)	<0.001
Male (%)	566 (14.1)	612 (15.2)	655 (16.3)	592 (14.7)	<0.001
BMI (kg/m^2^)	22 (20.1–23.6)	26.6 (25.2–28)	30.5 (28.9–32.4)	38 (34.8–43.4)	<0.001
HR	87.5 (74–103)	89 (74–105)	89 (77–105)	92 (79–108)	<0.001
SBP	123 (106–144)	125 (109–142)	125.76 (109–143)	123.5 (106–141)	0.028
DBP	70 (59–83)	70 (59–83)	71 (60–84)	69 (58–82)	0.178
Mean BP	83 (72–97)	84 (72–99)	85 (73–99)	83 (70–96)	0.559
**Laboratory tests**					
WBC (K/uL)	10.6 (7.5–14.6)	11 (7.8–15.4)	11.9 (8.6–16.2)	12.55 (8.8–16.8)	<0.001
RBC (K/uL)	3.64 (3.08–4.19)	3.76 (3.15–4.26)	3.85 (3.16–4.46)	3.9 (3.3–4.43)	<0.001
Hemoglobin (g/dL)	11 (9.3–12.6)	11.27 (9.6–12.9)	11.4 (9.6–13.4)	11.45 (9.8–13.1)	<0.001
Platelets (K/uL)	198 (148–273)	190 (133–256)	198 (139–251)	199 (148–266)	<0.001
Sodium (mEq/L)	139 (136–142)	139 (136–142)	139 (136–142)	138 (135–141)	0.101
Potassium (mEq/L)	4.0 (3.6–4.4)	4.1 (3.7–4.5)	4.1 (3.7–4.6)	4.2 (3.8–4.8)	<0.001
Calciumtotal (mEq/L)	8.3 (7.7–8.8)	8.4 (7.8–8.9)	8.3 (7.7–8.8)	8.3 (7.7–8.8)	0.032
TG (mg/dL)	91 (69–130)	115 (85–161)	147 (103–218)	195 (126–325)	<0.001
FPG (mg/dl)	114 (97–143)	127 (104–160)	137 (112–181)	156 (122–211)	<0.001
BUN (mg/dL)	18 (12–28)	19 (13–29)	19 (14–33)	22 (15–37)	<0.001
Creatinine (mg/dL)	0.9 (0.7–1.3)	1.0 (0.8–1.4)	1.1 (0.8–1.6)	1.2 (0.8–2.0)	<0.001
eGFR (mL/min/1.73 m^2^)	83.68 (53.08–104.03)	74.53 (47.44–96.56)	71.59 (44.18–96.56)	63.28 (33.86–91.87)	<0.001
**Comorbidities**					
HT	392 (9.7)	430 (10.7)	448 (11.1)	464 (11.5)	0.009
HF	259 (6.4)	254 (6.3)	263 (6.5)	289 (7.2)	0.292
MI	92 (2.3)	131 (3.3)	151 (3.8)	123 (3.1)	<0.001
Stroke	125 (3.1)	127 (3.2)	95 (2.4)	91 (2.3)	0.010
DM	155 (3.9)	221 (5.5)	294 (7.3)	429 (10.7)	<0.001
Sepsis	659 (16.4)	673 (16.7)	718 (17.8)	787 (19.6)	<0.001
Cancer	134 (3.3)	144 (3.6)	98 (2.4)	68 (1.7)	<0.001
**Score**					
SIRS	3 (2–3)	3 (2–3)	3 (2–3)	3 (2–4)	<0.001
SOFA	4 (2–7)	4.5 (2–8)	5 (2–9)	6 (3–10)	<0.001
SAPSII	36 (28–46)	35 (27–46)	37 (27–49)	39 (29–50)	<0.001
APSIII	44 (32–59)	44 (31–59)	46 (32–65)	52 (37–71)	<0.001
**Outcomes**					
AKI	758 (18.8)	816 (20.3)	880 (21.9)	920 (22.9)	<0.001
Early-onset AKI	510 (12.7)	578 (14.4)	670 (16.7)	777 (19.3)	<0.001
Late-onset AKI	248 (6.2)	238 (5.9)	210 (5.2)	143 (3.6)	<0.001

BMI, body mass index; HR, heart rate; SBP, systolic blood pressure; DBP, diastolic blood pressure; Mean BP, mean blood pressure; WBC, white blood cells; RBC, red blood cells; TG, triglycerides; FBG, fasting blood glucose; BUN, blood urea nitrogen; eGFR, estimated glomerular filtration rate; HT, hypertension; HF, heart failure; MI, myocardial infarction; DM, diabetes mellitus; SIRS, systemic inflammatory response syndrome; SOFA, Sequential Organ Failure Assessment; SAPSII, Simplified Acute Physiology Score II; APSIII, Acute Physiology Score III.

### TyG-BMI and early-onset AKI

**FIGURE 1:**
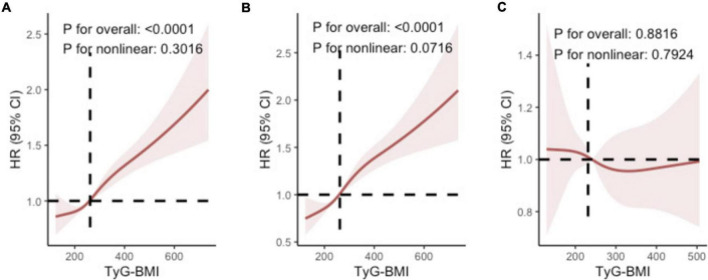
Restricted cubic spline analysis of the dose-response relationship between TyG-BMI and **(A)** overall AKI, **(B)** early-onset AKI, and **(C)** late-onset AKI.

Multivariable-adjusted HRs for early-onset AKI increased across TyG-BMI quartiles. Compared to Q1 (reference group), the HRs for Q2, Q3, and Q4 were 1.08 (95% CI: 0.96–1.22), 1.20 (95% CI: 1.06–1.34), and 1.40 (95% CI: 1.25–1.58), respectively (p for trend < 0.001; Table 2). Furthermore, each SD increase in TyG-BMI was associated with a 12% higher risk of early-onset AKI (HR 1.12 [95% CI: 1.08–1.17]). RCS analysis further confirmed the dose-response relationship between TyG-BMI and the risk of early-onset AKI (overall *p* < 0.0001), with an approximately linear increase when TyG-BMI exceeded 261.84 (non-linear *p* = 0.0716). In contrast, RCS analysis revealed no significant association between TyG-BMI and late-onset AKI (overall *p* = 0.8816, non-linear *p* = 0.7924; Figure 1). TyG-BMI levels were also positively correlated with early-onset AKI severity. Compared to Q1, the ORs for Q2, Q3, and Q4 were 1.55 (95% CI: 1.10–2.17), 1.64 (95% CI: 1.17–2.31), and 2.89 (95% CI: 1.94–4.30), respectively, for progression from AKI Stage 1 to Stage 2, and 1.56 (95% CI: 1.06–2.29), 1.90 (95% CI: 1.30–2.78), and 5.37 (95% CI: 3.52–8.19), respectively, for progression from AKI Stage 2 to Stage 3 (all p for trend < 0.001; Table 3).

**TABLE 2:** Cox proportional hazard ratios (HR) for early-onset AKI (≤48 h) across categories of TyG-BMI.

Outcome		Model 1	Model 2	Model 3
AKI	Q1 (Reference)	1	1	1
	Q2 (HR, 95% CI)	1.10 (0.98–1.24)	1.09 (0.96–1.23)	1.08 (0.96–1.22)
	Q3 (HR, 95% CI)	1.23 (1.09–1.38)	1.21 (1.07–1.36)	1.20 (1.06–1.34)
	Q4 (HR, 95% CI)	1.45 (1.30–1.62)	1.41 (1.26–1.59)	1.40 (1.25–1.58)
	P for trend	<0.001	<0.001	<0.001
	Per SD increase	1.13 (1.09–1.17)	1.12 (1.08–1.17)	1.12 (1.08–1.17)

Early-onset AKI was defined as AKI occurring within 48 h after ICU admission. Model 1 was unadjusted. Model 2 was adjusted for age, sex and eGFR. Model 3 was adjusted for all variables in model 2 plus SOFA, SAPSII and comorbidities (HT, HF, MI, Stroke, DM, Sepsis and Cancer).

**TABLE 3:** Association between TyG-BMI and severity of early-onset AKI (≤48 h) in critically ill patients.

TyG-BMI	AKI Stage 2 vs. AKI Stage 1	*P*-value	AKI Stage 3 vs. AKI Stage 2	*P*-value
	Adjusted OR (95% CI)		Adjusted OR (95% CI)	
Q1 (Reference)	1		1	
Q2 (HR, 95% CI)	1.55 (1.10–2.17)	0.010	1.56 (1.06–2.29)	0.022
Q3 (HR, 95% CI)	1.64 (1.17–2.31)	0.004	1.90 (1.30–2.78)	<0.001
Q4 (HR, 95% CI)	2.89 (1.94–4.30)	<0.001	5.37 (3.52–8.19)	<0.001
P for trend	<0.001		<0.001	

OR was adjusted for age, sex, eGFR, SOFA, SAPSII and comorbidities (HT, HF, MI, Stroke, DM, Sepsis and Cancer). AKI Stage 2 vs. AKI Stage 1 represents the risk of progression from Stage 1 to Stage 2, while AKI Stage 3 vs. AKI Stage 2 represents the risk of progression from Stage 2 to Stage 3.

### Subgroup analyses

Subgroup analyses demonstrated consistent associations between TyG-BMI and early-onset AKI risk across various population strata, including age, sex, eGFR, and comorbidities (e.g., hypertension, heart failure, DM, and SOFA scores) (all *p* < 0.05; Supplementary Table 1). These associations remained statistically significant across all strata and were particularly pronounced among males, younger patients (<60 years), individuals with preserved eGFR (≥90 mL/min/1.73 m^2^), and patients with DM, sepsis, or without prior hypertension or heart failure. Additionally, we performed ROC analyses for each subgroup to determine their optimal TyG-BMI cutoff values (Supplementary Table 2). The overall population had an optimal cutoff of 252.50, with most subgroups showing similar values. Notably, younger patients (<60 years: 280.50), males (284.50), and diabetic patients (283.50) had higher cutoffs, while patients with preserved renal function (eGFR ≥ 90: 216.50) had a lower cutoff. To identify extremely high-risk populations with multiple risk factors, we conducted stratified and interaction analyses. Stratified analysis showed that patients with high TyG-BMI and sepsis had the highest risk for early-onset AKI (HR = 2.42, 95% CI: 2.09–2.79), while those with high TyG-BMI and diabetes had HR = 1.31 (95% CI: 1.17–1.47) (Supplementary Tables 3, 4). Interaction analysis demonstrated significant TyG-BMI × sepsis interaction (per SD HR = 1.95, *p* < 0.001), modest TyG-BMI × diabetes interaction (per SD HR = 1.07, *p* < 0.05), and no significant TyG-BMI × DM × Sepsis interaction (per SD HR = 1.06, *p* > 0.05) (Supplementary Table 5).

### Predictive performance

Receiver operating characteristic curve analysis showed that TyG-BMI had an AUC of 0.661 (95% CI: 0.638–0.684; Supplementary Figure 1) for early-onset AKI, which was superior to its predictive ability for overall AKI (0.637 [95% CI: 0.615–0.660]) and late-onset AKI (0.566 [95% CI: 0.537–0.595]). DeLong’s test confirmed significant difference between early-onset and late-onset AKI prediction (*p* < 0.001). The optimal cutoff value for early-onset AKI was 252.50, with sensitivity of 0.604 (95% CI: 0.585–0.623), specificity of 0.648 (95% CI: 0.610–0.683), positive predictive value of 0.870 (95% CI: 0.853–0.885), negative predictive value of 0.296 (95% CI: 0.273–0.320), and Youden index of 0.252 (Table 4). NRI analysis showed that adding TyG-BMI to a base model (age and baseline serum creatinine) improved prediction for early-onset AKI (NRI = 0.141, 95% CI: 0.024–0.207), while showing no significant improvement for overall AKI (NRI = 0.069, 95% CI: −0.004 to 0.146) or late-onset AKI (NRI = −0.064, 95% CI: −0.155 to 0.287) (Supplementary Table 6). Decision curve analysis for early-onset AKI demonstrated increased net benefit with the enhanced model (including TyG-BMI) compared to the base model across various threshold probabilities (Supplementary Figure 2).

**TABLE 4:** Diagnostic performance and optimal threshold analysis of TyG-BMI for predicting AKI at different time windows.

Outcome	AUC (95% CI)	Optimal cutoff	Sensitivity (95% CI)	Specificity (95% CI)	PPV (95% CI)	NPV (95% CI)	Youden index	*P*-value
Overall AKI	0.637 (0.615–0.660)	252.50	0.568 (0.551–0.584)	0.648 (0.610–0.683)	0.893 (0.879–0.906)	0.224 (0.206–0.243)	0.215	
Early-onset AKI	0.661 (0.638–0.684)	252.50	0.604 (0.585–0.623)	0.648 (0.610–0.683)	0.870 (0.853–0.885)	0.296 (0.273–0.320)	0.252	0.1452^a^
Late-onset AKI	0.566 (0.537–0.595)	251.50	0.468 (0.435–0.502)	0.640 (0.602–0.676)	0.627 (0.588–0.664)	0.483 (0.449–0.516)	0.108	<0.0001^b,c^

AUC, Area Under the Receiver Operating Characteristic Curve; PPV, Positive Predictive Value; NPV, Negative Predictive Value; CI, Confidence Interval. *P*-values from DeLong test for AUC comparisons:

^a^Comparing Early-onset AKI vs. Overall AKI;

^b^Comparing Late-onset AKI vs. Overall AKI;

^c^Comparing Late-onset AKI vs. Early-onset AKI.

## Discussion

In this retrospective study of 4,024 critically ill patients from the MIMIC-IV database, we identified three key findings. First, TyG-BMI was confirmed as an independent predictor of early-onset AKI, with patients in the highest quartile demonstrating significantly increased risk compared to the lowest quartile (HR 1.40, 95% CI: 1.25–1.58), and each standard deviation increase in TyG-BMI associated with a 12% higher risk (HR 1.12, 95% CI: 1.08–1.17). Integration of TyG-BMI into a baseline model containing age and serum creatinine significantly improved risk reclassification for early-onset AKI, and decision curve analysis further confirmed clinical net benefit across various threshold probabilities for predicting early-onset AKI. Second, RCS analysis revealed the statistical pattern of risk change, with risk beginning to show an approximately linear increase when TyG-BMI exceeded 261.84, indicating a clear threshold for risk elevation. Finally, the optimal clinical cutoff value determined by the Youden index for early-onset AKI prediction was 252.50 (sensitivity 0.604, specificity 0.648), providing a more operationally practical reference point for risk assessment in actual clinical application. Collectively, these findings support the potential value of TyG-BMI as a clinical reference standard for early-onset AKI risk assessment in the ICU setting.

### TyG-BMI as a prognostic indicator of early-onset AKI

The TyG-BMI index has been validated as a reliable surrogate marker of insulin resistance (15). In insulin-resistant states, impaired IRS-1/PI3K/Akt metabolic signaling triggers compensatory hyperinsulinemia. While metabolic pathways remain blocked, hyperinsulinemia selectively activates renal growth pathways, promoting cellular hypertrophy and fibrosis via redox-sensitive kinases (ERK, JNK, mTOR/S6K1) (21). Concurrently, hyperinsulinemia downregulates miR-21 expression in glomerular endothelial cells, activating the MAPK/ET-1 pathway and inhibiting the PTEN/AKT/eNOS pathway, resulting in increased endothelin-1 production and decreased nitric oxide secretion, which causes vasoconstriction and endothelial dysfunction (22). Furthermore, impaired PI3K/Akt signaling reduces eNOS activation, disrupts tubuloglomerular feedback and promotes glomerular hyperfiltration and sodium retention. The synergistic activation of the sympathetic nervous system and renin-angiotensin-aldosterone system further amplifies these pathological effects. The combined effects of these mechanisms–endothelial dysfunction, oxidative stress, inflammation, and fibrosis–ultimately impair renal microcirculation and accelerate kidney injury progression (21). These mechanistic studies provide a solid pathophysiological foundation for the association between elevated TyG-BMI (reflecting insulin resistance) and increased AKI risk.

Building on this mechanistic understanding, our study further demonstrates that TyG-BMI shows a significant association with early-onset AKI risk in critically ill patients, while no significant association was observed with late-onset AKI. This temporal specificity can be attributed to multiple pathophysiological mechanisms: early-onset AKI typically originates from metabolic abnormalities and comorbidities present before ICU admission, whereas late-onset AKI is predominantly caused by iatrogenic factors, nosocomial infections, or complications developing during ICU stay (23–26). TyG-BMI incorporates the TyG index (a validated surrogate marker of insulin resistance) with BMI (reflecting overall adiposity), providing a comprehensive assessment of metabolic health that captures multiple pathophysiological pathways potentially involved in kidney injury. Insulin resistance (reflected by TyG) impairs renal function by disrupting glomerular hemodynamics and activating the renin-angiotensin system (27, 28); elevated triglycerides induce renal lipotoxicity through cellular damage and inflammatory signaling (29–31); while increased adiposity (reflected by BMI) amplifies these pathological processes through adipokine dysregulation and chronic inflammation (32, 33). In the critically ill state, stress responses further exacerbate these metabolic disturbances, specifically influencing the development of early-onset AKI. Consequently, TyG-BMI specifically predicts early-onset rather than late-onset AKI.

### Enhanced predictive value of TyG-BMI in specific populations

The association between TyG-BMI and early-onset AKI was more pronounced in specific subpopulations: males, younger patients (<60 years), individuals with preserved eGFR (≥90 mL/min/1.73 m^2^), and patients with diabetes or sepsis. These differential effects can be attributed to several physiological mechanisms. Males typically exhibit greater visceral adiposity, which is more metabolically active and pro-inflammatory, potentially amplifying the impact of metabolic dysregulation on renal function (34–36). While elderly patients generally exhibit a higher overall risk for AKI (37), the stronger predictive performance of TyG-BMI in younger individuals aligns with previous research showing that the ability to predict AKI risk using established risk factors tends to decline with advancing age (38). This age-dependent difference may reflect the increasing complexity of renal pathophysiology in older adults, where multiple comorbidities and age-related structural changes may overshadow metabolic factors as primary drivers of kidney injury. In contrast, younger individuals with fewer competing risk factors may exhibit more direct associations between metabolic dysregulation and kidney injury. In diabetic patients, underlying microvascular damage and metabolic dysfunction predispose the kidneys to further injury, particularly as chronic insulin resistance may synergistically interact with acute metabolic derangements to accelerate nephrotoxic processes (39–41). Our stratified and interaction analyses revealed that sepsis showed the strongest interaction with TyG-BMI. Patients with high TyG-BMI and sepsis demonstrated the highest risk (HR = 2.42) with strong synergistic effects (interaction term per SD HR = 1.95), indicating that metabolic dysfunction and sepsis-induced inflammatory responses amplify each other to accelerate kidney injury (42). Notably, the three-way interaction (TyG-BMI × DM × Sepsis) was not significant (per SD HR = 1.06, *p* > 0.05), suggesting that the presence of diabetes does not further amplify the already strong TyG-BMI-sepsis interaction. Collectively, these findings emphasize the particular value of TyG-BMI for risk stratification in these specific populations, supporting the implementation of individualized monitoring and preventive strategies for these susceptible groups.

### TyG-BMI thresholds as clinical reference standards for AKI risk

This study established a linear dose-response relationship between TyG-BMI and AKI risk, revealing differentiated threshold patterns across various AKI onset windows. For both overall AKI and early-onset AKI, the risk curves clearly demonstrated that HRs began to show an approximately linear increase beyond the threshold of 261.84; whereas for late-onset AKI, no statistically significant association was observed between TyG-BMI and risk.

Through Youden index analysis, we identified that overall AKI and early-onset AKI share a consistent optimal clinical threshold of 252.50, highlighting the clinical universality of this cutoff value. This threshold precisely balances sensitivity (0.604) and specificity (0.648) for predicting early-onset AKI, while also providing comparable predictive performance for overall AKI (sensitivity 0.568, specificity 0.648). Importantly, our subgroup analyses revealed that while this 252.50 threshold remained applicable for most clinical subgroups, certain populations showed notable variations. Patients with preserved renal function (eGFR ≥ 90) had a lower optimal threshold (216.50), suggesting heightened sensitivity to metabolic abnormalities, whereas diabetic patients (283.50) and younger males (280.50) required higher thresholds, likely reflecting their elevated baseline metabolic burden. Despite these variations, TyG-BMI maintained good predictive performance across all subgroups (AUC > 0.60), supporting its universal applicability as an AKI risk assessment tool.

Notably, our study also revealed a significant association between TyG-BMI and AKI severity. The ORs for progression from Stage 1 to Stage 2 AKI increased dramatically across TyG-BMI quartiles (ORs for Q2, Q3, and Q4 were 1.55, 1.64, and 2.89, respectively), with an even more pronounced effect for progression from Stage 2 to Stage 3 (ORs of 1.56, 1.90, and 5.37). This gradient effect suggests that TyG-BMI not only predicts AKI occurrence but also its severity, potentially reflecting how the degree of metabolic dysfunction influences the extent of kidney injury. The particularly strong association with Stage 3 AKI (over 5-fold increased risk in the highest quartile) highlights TyG-BMI’s value in identifying patients at risk for the most severe forms of AKI, which carry the highest morbidity and mortality.

Consequently, at the clinical practice level, while 252.50 serves as a valuable initial screening threshold, physicians should consider patient-specific adjustments: using lower thresholds (216.50) for patients with preserved renal function to enable early risk identification, and higher thresholds for younger males (280.50) or diabetic patients (283.50) to avoid overdiagnosis. For patients exceeding their respective thresholds, physicians should implement more proactive preventive strategy combinations, including intensified renal function monitoring protocols, refined fluid management strategies, and judicious use of potentially nephrotoxic medications. Future research directions should focus on validating these population-specific thresholds in diverse clinical populations and systematically exploring the potential impact of targeted early interventions on improving AKI-related clinical outcomes.

### Future perspectives: implementing TyG-BMI in critical care settings

Implementing TyG-BMI as a routine tool for AKI assessment in critical care environments offers significant clinical application prospects. TyG-BMI provides a simple, cost-effective early risk stratification tool that can be easily calculated from laboratory values routinely obtained at ICU admission. From a practical perspective, clinicians can calculate TyG-BMI upon admission to identify patients at high risk for early-onset AKI, with particular attention to those exceeding our established optimal cutoff value of 252.50. This early identification may trigger a series of preventive interventions, including more intensive monitoring of renal function, cautious use of nephrotoxic medications, careful fluid management, and early nephrology consultation.

Furthermore, since the components of TyG-BMI are potentially modifiable, targeted interventions warrant further elaboration. Studies have shown that intensive insulin therapy in surgical ICU patients can reduce AKI incidence (43); hypertriglyceridemia has been confirmed to be associated with increased risk of AKI (44); and obesity is an independent risk factor for AKI development (45). For patients with high TyG-BMI, aggressive glycemic control within the first 48 h after admission, nutritional adjustments to limit lipid intake, and optimized fluid management to avoid volume overload may reduce the risk of early-onset AKI. However, given the interactions and synergistic effects among blood glucose, lipids, and BMI, the optimal strategy for high TyG-BMI patients may be an integrated intervention protocol that simultaneously optimizes all three parameters. While our study establishes TyG-BMI as a predictor of early-onset AKI, prospective randomized controlled trials are needed to validate the effectiveness of these specific interventions and determine optimal implementation strategies. For healthcare systems, implementing TyG-BMI screening can help allocate resources more efficiently by focusing preventive efforts on patients at highest risk for early renal complications. This risk stratification approach may reduce unnecessary testing and interventions while ensuring high-risk patients receive appropriate attention, thereby optimizing resource utilization and improving patient safety.

### Strengths and limitations

This study possesses several notable strengths, including the utilization of a large comprehensive critical care database (MIMIC-IV) with detailed clinical information, laboratory data, and outcome assessments for the included patients. We employed multiple rigorous statistical methodologies to comprehensively evaluate the association between TyG-BMI and AKI. The extensive subgroup analyses across various clinically relevant populations further enhanced the generalizability of our findings. Additionally, TyG-BMI can be easily calculated from routinely obtained laboratory values, demonstrating excellent clinical feasibility and practical utility, requiring no additional testing for daily risk assessment, thus making it an ideal risk stratification tool in critical care environments.

Despite these strengths, several limitations warrant consideration. First, the retrospective, observational design precludes definitive causal inference regarding the relationship between TyG-BMI and AKI. Second, although we extensively adjusted for potential confounding factors, residual confounding may still persist. Third, the MIMIC-IV database reflects the patient population and clinical practices of a single academic medical center, potentially limiting generalizability to other settings. Fourth, our inclusion criteria requiring triglyceride, glucose, and BMI measurements may have introduced selection bias, as these parameters are more likely to be measured in patients with suspected metabolic abnormalities. Consequently, future research directions should include prospective, multicenter validation studies to confirm our findings across diverse ICU populations.

## Conclusion

In conclusion, this study demonstrates that TyG-BMI serves as an independent predictor of early-onset AKI in critically ill patients and provides significant incremental value when incorporated into traditional risk assessment models. The identification of a specific TyG-BMI threshold (252.50) offers an evidence-based reference point for risk stratification in clinical practice. The consistent association between TyG-BMI and early-onset AKI across various subpopulations, with particularly pronounced signals in males, younger patients (<60 years), individuals with preserved eGFR (≥90 mL/min/1.73 m^2^), and patients with diabetes or sepsis, supports the development of targeted preventive strategies for these high-risk groups. Future prospective studies are warranted to validate these findings and evaluate the clinical efficacy of preventive interventions guided by TyG-BMI assessment.

## Data Availability

Publicly available datasets were analyzed in this study. This data can be found here: the data used in this study are available in the MIMIC-IV clinical database (version 3.0), which is accessible through the PhysioNet repository (https://physionet.org/content/mimiciv/3.0/). The database has a PhysioNet accession number: mimiciv/3.0. Access to this repository requires completion of the CITI “Data or Specimens Only Research” course and acceptance of the data use agreement. The specific code used for data extraction and analysis in this study is available from the corresponding author upon reasonable request.

## Footnotes

1
https://physionet.org/content/mimiciv/
