# Supplementary material for: Triglyceride-glucose-body mass index predicts early-onset acute kidney injury in critically ill patients: a retrospective analysis using the MIMIC-IV database
Source: Front Nutr. 2026 Jan 12;12:1721579. doi: 10.3389/fnut.2025.1721579 (PMC12832482; doi:10.3389/fnut.2025.1721579)
Supplement: Supplementary file 1 [file Data_Sheet_1.docx]

**Table S1. Subgroup analyses of hazard ratios (HRs) for early-onset AKI (≤48h) across categories of TyG-BMI.**

|  | AKI rate | | TyG-BMI | | | | | | P Value | | P Value for interaction | |  |  |
| --- | --- | --- | --- | --- | --- | --- | --- | --- | --- | --- | --- | --- | --- | --- |
|  | no. of events/total no. (%) | |  |  |  |  |  |  |  |  |  |  |  |  |
| 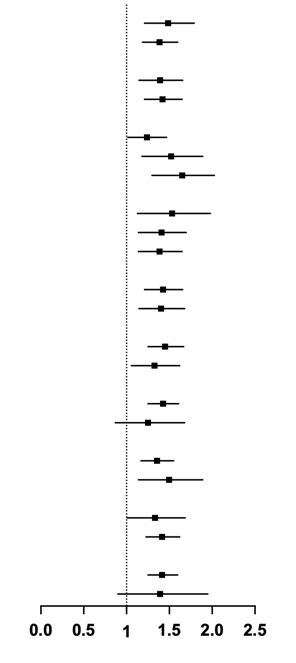Age | | | | | | | | | | | | < 0.001 | |  |
| < 60 | | 946/1599 (59.2)  1589/2425 (65.5) | | |  | 1.47 (1.21-1.79) | | | | < 0.001 | |  |  |  |
| $\geq$ 60 | |  |  |  |  | 1.38 (1.19-1.60) | | | | < 0.001 | |  |  |  |
| Sex | | | | | | | | | | | | 0.002 | |  |
| Female | | 977/ 1599 (61.1)  1558/2425 (64.2) | |  | | 1.38 (1.15-1.66) | | | | < 0.001 | |  |  |  |
| Male | |  |  |  |  | 1.41 (1.21-1.65) | | | | < 0.001 | |  |  |  |
| eGFR | | | | | | | | | | | | 0.016 | |  |
| ＜60 | | 1099/1526 (72.0) 730/1127 (64.8)  705/1369 (51.5) | |  | | 1.23 (1.02-1.47) | | | | 0.024 | |  | | |
| 60-89 | |  |  |  |  | 1.49 (1.18-1.89) | | | | < 0.001 | |  | | |
| ≥90 | |  |  |  |  | 1.62 (1.30-2.03) | | | | < 0.001 | |  | | |
| SOFA score | | | | | | | | | | | | < 0.001 | |  |
| ＜3 | | 520/1030 (50.5)  874/1441 (60.7) | |  | | 1.50 (1.13-1.98) | | | | 0.004 | |  | | |
| 3-7 | |  |  |  |  | 1.39 (1.14-1.70) | | | | 0.001 | |  | | |
| ≥7  History of HT  No  Yes  History of HF  NO  Yes  History of MI or Stroke  NO  Yes  History of DM  No  Yes  History of Sepsis  No  Yes | | 1141/1553 (73.5)  1427/2290 (62.3)  1108/1734 (63.9)  1783/2959 (60.3)  752/1065 (70.6)  2178/3527 (61.8)  357/497 (71.8)  1806/2925 (61.7)  729/1099 (66.3)  605/1187 (51.0)  1930/2837 (68.0) | |  | | 1.37 (1.14-1.65)  1.42 (1.21-1.66)  1.39 (1.15-1.68)  1.44 (1.25-1.67)  1.31 (1.06-1.62)  1.42 (1.25-1.61)  1.21 (0.87-1.68)  1.35 (1.17-1.55)  1.47 (1.14-1.89)  1.31 (1.01-1.69)  1.41 (1.23-1.62) | | | | < 0.001  < 0.001  < 0.001  < 0.001  0.012  < 0.001  0.239  < 0.001  0.003  0.035  < 0.001 | | 0.337  0.001  0.023  0.006  0.053 | | |
| History of Cancer | | | | | | | | | | | | < 0.001 | |  |
| No | | 2268/3580 (63.4)  267/444 (60.1) | |  | | 1.41 (1.25-1.60) | | | | < 0.001 | |  | | |
| Yes | |  |  |  |  | 1.33 (0.90-1.95) | | | | 0.145 | |  | | |
|  | |  | |  | | |  |  | | | |  | | |

**Table S2. Subgroup-Specific Diagnostic Performance and Optimal Threshold Analysis of TyG-BMI for Predicting Early-onset AKI.**

| Subgroup | AUC (95% CI) | Optimal Cutoff | Sensitivity (95% CI) | | Specificity (95% CI) | PPV (95% CI) | NPV (95% CI) | Youden Index |
| --- | --- | --- | --- | --- | --- | --- | --- | --- |
| Age＜60 | 0.663 (0.629-0.698) | 280.50 | | 0.514 (0.482-0.545) | 0.742 (0.690-0.788) | 0.860 (0.829-0.886) | 0.330 (0.296-0.366) | 0.256 |
| Age≥60 | 0.671 (0.642-0.701) | 252.50 | | 0.571 (0.546-0.595) | 0.709 (0.659-0.755) | 0.901 (0.881-0.918) | 0.263 (0.236-0.293) | 0.280 |
| Female | 0.687 (0.654-0.720) | 250.50 | | 0.596 (0.565-0.626) | 0.699 (0.643-0.750) | 0.874 (0.846-0.897) | 0.331 (0.294-0.369) | 0.295 |
| Male | 0.639 (0.608-0.670) | 284.50 | | 0.442 (0.418-0.467) | 0.787 (0.743-0.826) | 0.897 (0.874-0.917) | 0.252 (0.227-0.277) | 0.229 |
| eGFR＜60 | 0.670 (0.623-0.716) | 253.50 | | 0.638 (0.609-0.666) | 0.657 (0.573-0.732) | 0.938 (0.919-0.954) | 0.181 (0.149-0.218) | 0.295 |
| eGFR 60-89 | 0.682 (0.642-0.722) | 252.50 | | 0.608 (0.572-0.643) | 0.704 (0.635-0.764) | 0.888 (0.857-0.913) | 0.317 (0.275-0.363) | 0.312 |
| eGFR≥90 | 0.617 (0.581-0.654) | 216.50 | | 0.771 (0.738-0.800) | 0.425 (0.373-0.479) | 0.743 (0.710-0.773) | 0.462 (0.406-0.518) | 0.196 |
| No HT | 0.670 (0.639-0.701) | 254.50 | | 0.580 (0.554-0.606) | 0.689 (0.639-0.736) | 0.884 (0.862-0.903) | 0.288 (0.258-0.319) | 0.270 |
| HT | 0.653 (0.619-0.686) | 256.50 | | 0.605 (0.576-0.633) | 0.632 (0.576-0.685) | 0.859 (0.833-0.882) | 0.301 (0.267-0.338) | 0.237 |
| No HF | 0.663 (0.637-0.688) | 254.50 | | 0.598 (0.575-0.621) | 0.648 (0.607-0.687) | 0.847 (0.827-0.866) | 0.330 (0.303-0.359) | 0.246 |
| HF | 0.660 (0.611-0.710) | 255.50 | | 0.581 (0.546-0.616) | 0.714 (0.622-0.792) | 0.936 (0.910-0.955) | 0.192 (0.156-0.234) | 0.295 |
| No DM | 0.656 (0.630-0.682) | 251.50 | | 0.551 (0.528-0.574) | 0.688 (0.645-0.727) | 0.867 (0.847-0.886) | 0.292 (0.267-0.319) | 0.239 |
| DM | 0.664 (0.617-0.710) | 283.50 | | 0.624 (0.588-0.659) | 0.687 (0.612-0.753) | 0.899 (0.870-0.923) | 0.290 (0.247-0.337) | 0.311 |

**Table S3. Cox proportional hazard ratios (HR) for early-onset AKI (≤48h) by TyG-BMI and diabetes status.**

| Outcome | Group | HR (95% CI) |
| --- | --- | --- |
| Early-onset AKI | Low TyG-BMI without DM (Reference) | 1 |
|  | Low TyG-BMI with DM | 0.87 (0.74-1.03) |
|  | High TyG-BMI without DM | 1.36 (1.23-1.50) |
|  | High TyG-BMI with DM | 1.31 (1.17-1.47) |

HR was adjusted for age, sex, eGFR, SOFA, SAPSII and comorbidities (HT, HF, MI, Stroke, Sepsis and Cancer).

High TyG-BMI was defined as TyG-BMI > 252.50, and low TyG-BMI as TyG-BMI ≤ 252.50 based on the optimal cutoff value determined by ROC curve analysis for predicting early-onset AKI.

**Table S4. Cox proportional hazard ratios (HR) for early-onset AKI (≤48h) by TyG-BMI and sepsis status.**

| Outcome | Group | HR (95% CI) |
| --- | --- | --- |
| Early-onset AKI | Low TyG-BMI without Sepsis (Reference) | 1 |
|  | Low TyG-BMI with Sepsis | 1.81 (1.56-2.09) |
|  | High TyG-BMI without Sepsis | 1.56 (1.33-1.83) |
|  | High TyG-BMI with Sepsis | 2.42 (2.09-2.79) |

HR was adjusted for age, sex, eGFR, SOFA, SAPSII and comorbidities (HT, HF, MI, Stroke, Sepsis and Cancer).

High TyG-BMI was defined as TyG-BMI > 252.50, and low TyG-BMI as TyG-BMI ≤ 252.50 based on the optimal cutoff value determined by ROC curve analysis for predicting early-onset AKI.

**Table S5. Interaction analysis between TyG-BMI and clinical factors for early-onset AKI (<48h).**

| Outcome |  | | Model S1 | Model S2 | Model S3 |
| --- | --- | --- | --- | --- | --- |
| Early-onset AKI | | Q1 (Reference) | 1 | 1 | 1 |
|  | | Q2 (HR, 95% CI) | 1.24 (0.72–2.13) | 1.52 (1.09–2.14) | 1.05 (0.49–2.24) |
|  | | Q3 (HR, 95% CI) | 4.55 (2.51–8.34) | 2.73 (1.86–4.07) | 4.28 (1.84–10.35) |
|  | | Q4 (HR, 95% CI) | 4.60 (2.60–8.21) | 1.41 (0.84–3.08) | 5.34 (2.47–12.70) |
|  | | P for trend | < 0.001 | < 0.001 | < 0.001 |
|  | | Per SD increase | 1.07 (1.01–1.18) | 1.95 (1.74–2.20) | 1.06 (1.00–1.17) |

Model S1: The interaction term TyG-BMI × DM, adjusted for age, sex, eGFR, SOFA, SAPSII, and comorbidities (HT, HF, MI, Stroke, Sepsis, and Cancer).

Model S2: The interaction term TyG-BMI × Sepsis, adjusted for age, sex, eGFR, SOFA, SAPSII, and comorbidities (HT, HF, MI, Stroke, DM, and Cancer).

Model S3: The interaction term TyG-BMI × DM × Sepsis, adjusted for age, sex, eGFR, SOFA, SAPSII, and comorbidities (HT, HF, MI, Stroke, and Cancer).

**Table S6. Comparison of Net Reclassification Improvement between Two Prognostic Models for AKI.**

| Outcome | Model | C-statistic (95% CI) | NRI (95% CI) |
| --- | --- | --- | --- |
| Overall AKI | Base | 0.554 (0.538-0.570) | Reference |
|  | Enhanced | 0.601 (0.586-0.622) | 0.069 (-0.004-0.146) |
|  |  |  |  |
| Early-onset AKI | Base | 0.593 (0.569-0.616) | Reference |
|  | Enhanced | 0.627 (0.603-0.651) | 0.141 (0.024-0.207) |
|  |  |  |  |
| Late-onset AKI | Base | 0.539 (0.512-0.566) | Reference |
|  | Enhanced | 0.536 (0.509-0.563) | -0.064 (-0.155-0.287) |

Model Base: Includes age and baseline serum creatinine as predictors.

Model Enhanced: Includes age, baseline serum creatinine and TyG-BMI as predictors.

NRI , Net Reclassification Improvement.


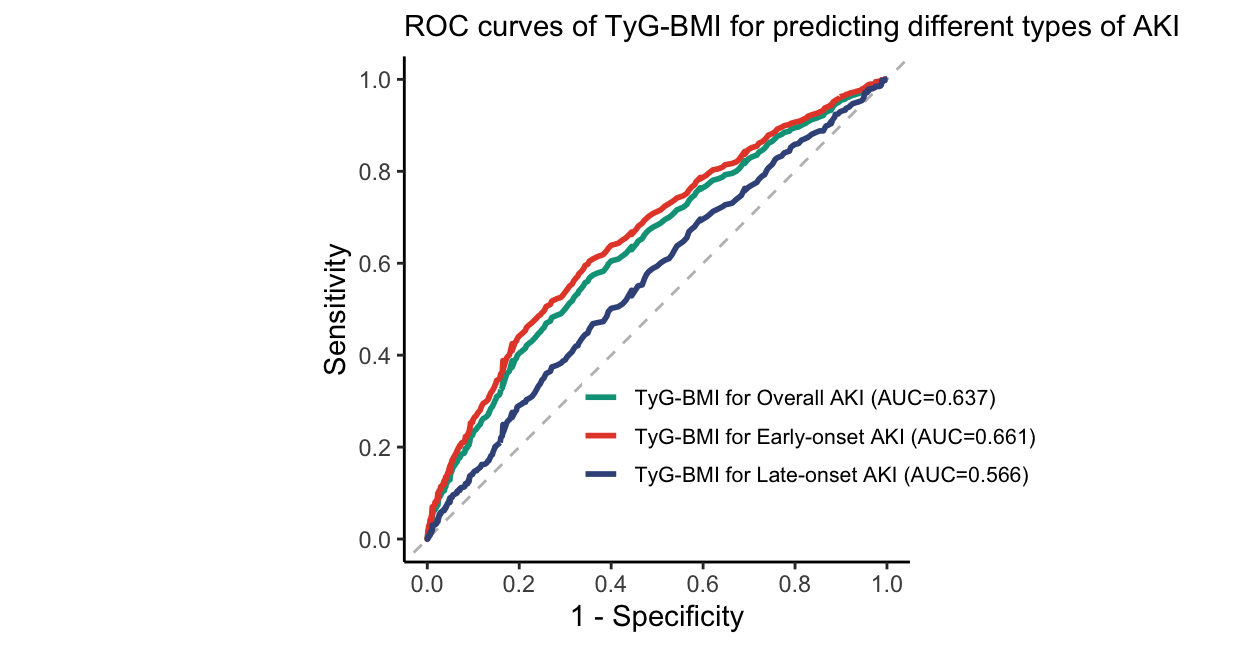


**Figure S1.** Receiver operating characteristic (ROC) curve analysis showing the comparative predictive performance of TyG-BMI for early-onset AKI, late-onset AKI, and overall AKI in critically ill patients.

| Performance Metrics | Base Model | Enhanced Model |
| --- | --- | --- |
| AIC | 38397.44 | 38308.48 |
| Residual Deviance | 38389.44 | 38298.48 |
| Prediction Probability Range | 0.423-0.977 | 0.335-0.979 |


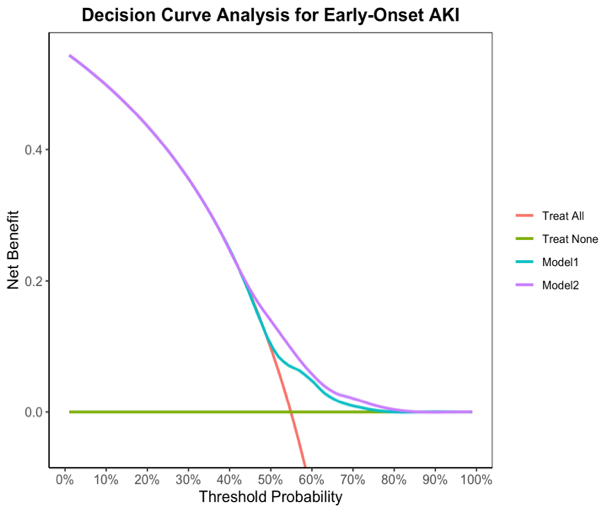


**Figure S2.** Decision curve analysis (DCA) comparing the clinical utility of different prediction models for early-onset acute kidney injury in critically ill patients.

The graph shows the net benefit across various threshold probabilities for the treat all strategy (red), treat none strategy (green), base model (blue) including age and baseline serum creatinine, and enhanced model (purple) incorporating age, baseline serum creatinine and TyG-BMI as predictors. The accompanying table shows that the enhanced model achieved better performance metrics, including lower AIC and residual deviance values, and a broader prediction probability range, particularly extending toward lower risk identification.
